# Enhanced Expression of Vacuolar H^+^-ATPase Subunit E in the Roots Is Associated with the Adaptation of *Broussonetia papyrifera* to Salt Stress

**DOI:** 10.1371/journal.pone.0048183

**Published:** 2012-10-25

**Authors:** Min Zhang, Yanming Fang, Zhenhai Liang, Libin Huang

**Affiliations:** 1 Jiangsu Academy of Forestry, Dongshanqiao, Jiangning, Nanjing, Jiangsu, China; 2 College of Forest Resources and Environment, Nanjing Forestry University, Nanjing, China; University College Dublin, Ireland

## Abstract

Vacuolar H^+^-ATPase (V-H^+^-ATPase) may play a pivotal role in maintenance of ion homeostasis inside plant cells. In the present study, the expression of V-H^+^-ATPase genes was analyzed in the roots and leaves of a woody plant, *Broussonetia papyrifera*, which was stressed with 50, 100 and 150 mM NaCl. Moreover, the expression and distribution of the subunit E protein were investigated by Western blot and immunocytochemistry. These showed that treatment of *B. papyrifera* with NaCl distinctly changed the hydrolytic activity of V-H^+^-ATPase in the roots and leaves. Salinity induced a dramatic increase in V-H^+^-ATPase hydrolytic activity in the roots. However, only slight changes in V-H^+^-ATPase hydrolytic activity were observed in the leaves. In contrast, increased H^+^ pumping activity of V-H^+^-ATPase was observed in both the roots and leaves. In addition, NaCl treatment led to an increase in H^+^-pyrophosphatase (V-H^+^-PPase) activity in the roots. Moreover, NaCl treatment triggered the enhancement of mRNA levels for subunits A, E and c of V-H^+^-ATPase in the roots, whereas only subunit c mRNA was observed to increase in the leaves. By Western blot and immunocytological analysis, subunit E was shown to be augmented in response to salinity stress in the roots. These findings provide evidence that under salt stress, increased V-H^+^-ATPase activity in the roots was positively correlated with higher transcript and protein levels of V-H^+^-ATPase subunit E. Altogether, our results suggest an essential role for V-H^+^-ATPase subunit E in the response of plants to salinity stress.

## Introduction

Plant cells are characterized by the presence of a large central vacuole in most differentiated tissues; the vacuole plays a crucial role in plants' tolerance to salinity [1], [2]. Two plant proton pumps, vacuolar H^+^-ATPase (V-H^+^-ATPase) and H^+^-pyrophosphatase (V-H^+^-PPase), participate in acidifying compartments of the vacuoles, which establishes an electrochemical H^+^-gradient to drive sequestration of Na^+^ into the vacuole lumen, compartmentalizing this toxic ion from the cytoplasm and maintaining low cytoplasmic Na^+^ concentrations [2], [3], [4]. V-H^+^-ATPase is an ATP-dependent proton pump that couples the energy released upon hydrolysis of ATP to the active transport of protons from the cytoplasm to the lumen of the intracellular compartment [5]. V-H^+^-ATPase is a multi-subunit complex organized into two distinct sectors. The first is the peripherally associated, hydrophilic V_1_ domain, which is composed of eight different subunits (A–H) and hydrolyzes ATP, and the second is the hydrophobic, membrane-anchored V_0_ domain consisting of six different subunits, which functions to translocate protons across the membrane [6], [7]. V-H^+^-PPase coexists with V-H^+^-ATPase in the vacuolar membrane, and together they are the major components of the vacuolar membrane in plant cells [4]. Unlike V-H^+^-ATPase, V-H^+^-PPase consists of only a single polypeptide and exists as a dimer of identical subunits [8].

Accumulating evidence has implicated the regulation of V-H^+^-ATPase activity by salt both in glycophytes and halophytes [9]–[11]. It was reported that in cell suspensions of *Populus euphratica*, V-H^+^-ATPase hydrolytic and H^+^ pumping activities were stimulated in response to salt stress [12]. The strategy of *Suaeda salsa* to adapt to high salinity seems to be an up-regulation of V-H^+^-ATPase activity [13]. The V-H^+^-ATPase hydrolytic and proton pump activity in tonoplast vesicles derived from the salt-treated leaves of *S. salsa* were significantly elevated compared to that of control leaves. Up-regulated activity of V-H^+^-ATPase has also been observed in cucumber [14] and *Vigna unguiculata* [15]. Regulation of V-H^+^-ATPase transport activity has been suggested to operate at the transcriptional level as well as the protein level under salt stress [16]–[18]. In the halotolerant sugar beet, an increase in mRNA was paralleled by an increase in the amount of V-H^+^-ATPase protein [19]. Contradictory reports have also claimed that the salt-mediated increase in V-H^+^-ATPase activity is not associated with an increase in protein expression [20], [21]. Despite the volume of studies on changes in V-H^+^-ATPase and plant salt tolerance to date, little is known about the correlation between activation of this proton pump and salt tolerance in woody plants.

*Broussonetia papyrifera*, a tree belonging to the Moraceae family, is important as a source of fiber for cloth and paper. The tree is a vigorous pioneer species, which can rapidly colonize forest clearings and is widely favored because of its fast growth [22]. *B. papyrifera* is tolerant to drought and resistant to salt stress, which makes it an ideal tree species to use for controlling salinity [23].

In the present study, we exploited RT-PCR and Western blot analysis as well as immunocytochemistry to investigate tissue-specific expression of V-H^+^-ATPase in the leaves and roots of the woody plant *B. papyrifera* in response to NaCl stress. In addition, the hydrolytic activities of V-H^+^-ATPase and V-H^+^-PPase were determined by spectrophotometric analysis, and proton pumping activity of V-H^+^-ATPase was assayed by monitoring the quenching of ACMA fluorescence. Moreover, vacuolar pH was examined using the fluorescent pH probe BCECF AM by laser scanning confocal microscopy.

## Materials and Methods

### Plant material and growth conditions

*In vitro* regenerated *B. papyrifera* rooting plantlets of uniform size were grown in plastic pots filled with 500 ml of 1/2MS solutions. All experiments were conducted under controlled conditions (light/dark cycle of 16/8 h at 25±2°C, illumination of 2000 Lx). Salinity treatments were initiated by adding NaCl to 1/2MS solution to achieve final concentrations of 50 mM, 100 mM or 150 mM. The nutrient solution was changed every other day. The roots and leaves were harvested five days after NaCl exposure. Unstressed plants grown in parallel served as the control and were harvested at the same time.

### Preparation of vacuolar membrane vesicles

Tonoplast-enriched vesicles were isolated according to the method of Giannini and Briskin [24] with some modifications. Fresh leaves or roots were homogenized in homogenization buffer (70 mM Tris/HCl, pH 8.0, 250 mM sucrose, 2 mM EDTA, 2 mM ATP-Na2, 1% BSA, 0.5% PVP-40, 4 mM DTE, 10% glycerol, 250 mM KCl) containing protease inhibitor cocktail (Roche, Indianapolis, IN, USA). The homogenate was centrifuged at 13,000 g at 4°C for 15 min, and the supernatant was then centrifuged at 80,000 g for 30 min in a Beckman 70Ti rotor. The membrane pellet was resuspended in 4 ml suspension buffer (2 mM BTP/Mes, pH 7.0, 250 mM sucrose, 0.2% BSA, 10% glycerol, 1 mM DTE) and layered over a 25/38% (w/w) discontinuous sucrose density gradient. After centrifuging at 100,000 g for 2 h in a Beckman Optima L-80XP ultracentrifuge with an SW 41Ti rotor, the vacuolar membrane vesicles were removed from the 8/25% interface and stored at −80°C. The membrane protein concentration was assayed by the method of Lowry et al [25], and bovine serum albumin was used as the protein standard.

### Assay of V-H^+^-ATPase and V-H^+^-PPase hydrolytic activities

The hydrolytic activities of V-H^+^-ATPase and V-H^+^-PPase were determined by measuring the amount of inorganic phosphate released [26]. The reaction was initiated by adding 20 µg of vacuolar membrane protein into the reaction buffer. For V-H^+^-ATPase, the reaction medium contained 25 mM Tris-Mes (pH 7.0), 4 mM MgSO_4_·7H_2_O, 50 mM KCl, 1 mM NaN_3_, 0.1 mM Na_2_MoO_4_, 0.1% Brij 35, 500 μM NaVO_4_, and 2 mM ATP-Na_2_, whereas the reaction medium for V-H^+^-PPase consisted of 25 mM Tris-Mes (pH 7.5), 2 mM MgSO_4_ H_2_O, 0.1 mM Na_2_MoO_4_, 0.1% Brij 58, and 0.2 mM K_4_P_2_O_7_. The reaction mixture was incubated at 28°C for 40 min, and then terminated by the addition of 3% TCA. Inorganic phosphate was assayed according to Ames [27]. For the determination of V-H^+^-ATPase activity, Pi release was measured in the presence and absence of 100 nM concanamycin A (specific inhibitor of V-H^+^-ATPase) and the difference between these two activities was attributed to V-H^+^-ATPase activity. And K^+^-dependent H^+^-PPase activity was calculated as the difference in activity in the presence and absence of 50 mM KCl. The enzymatic activities are presented in µmol Pi·mg^−1^protein·h^−1^.

### Proton pumping assay

The proton pumping activity of the isolated tonoplast vesicles was measured spectrophotometrically by monitoring the quenching of ACMA (9-amino-6-chloro-2-methoxyacridine) fluorescence as described previously with minor modification [28]. The assay buffer contained 10 mM Mes-Tris (pH 7.5), 250 mM sorbitol, 50 mM KCl, 3 mM ATP, 50 μM NaVO_4_, 1 mM NaN_3_ and 2 µM ACMA. The reaction was initiated by adding 3.5 mM MgSO_4_, and fluorescence quenching (415 nm excitation and 485 nm emission) was measured in a Hitachi 850 fluorescence spectrometer at 22 °C. Proton pumping activity was expressed as % quench mg^−1^ protein min^−1^.

### RT-PCR

Total RNA was extracted from fresh roots and leaves using TRIzol reagent (Invitrogen, Carlsbad, California, USA) according to the manufacturer's instructions. cDNA was synthesized with the GoScript™ Reverse Transcription System (Promega, Madison, Wisconsin) following the manufacturer's protocol. The resulting cDNA was then used as template for the PCR reaction. PCR cycling was performed with an ABI 2720 thermocycler (Applied Biosystems). The PCR program was as follows [17]: predenaturation at 94°C for 1.5 min and then a total of 25 cycles of denaturation at 94°C for 1 min, annealing at 55°C for 1 min and extension at 72°C for 2 min, followed by one cycle of final extension at 72°C for 10 min. PCR products were run on 1% agarose gels and stained with ethidium bromide. Primers used for the PCR reactions are shown in Table 1.

**Table 1:** Nucleotide sequence of the primers used in this study.

Subunit	Nucleotide sequence
**A**	5′-GATCCTGTTACATCTGCA-3′ 5′-AGACTGACTTGTAGAACG-3′
**B**	5′-GCTAGAGGGCAGAAGATT-3′ 5′-GTGGTGTGATGATACGCT-3′
**E**	5′-GAGAAGGCCACCGAGATC-3′ 5′-GCAACGCAACAAGACAGC-3′
**c**	5′-ACCGTCTTCAATGGCGAT-3′ 5′-CGACAATGAGACCGTAGA-3′
**actin**	5′-GTGATCTCCTTGCTCATACG-3′ 5′-GGNACTGGAATGGTNAAGG-3′

### Western blot analysis

Membrane proteins were separated by sodium dodecylsulfate polyacrylamide gel electrophoresis (SDS-PAGE) according to previously described procedures [29] and the separated proteins were electrophoretically transferred onto polyvinylidene difluoride (PVDF) membranes (Bio-Rad, Hercules, CA, USA). Subsequently, the membranes were blocked with 1% bovine serum (BSA) in Tris-buffered saline (TBS) for 20 min. The membranes were then incubated with a rabbit anti-VHA-E antibody from *Arabidopsis thaliana* (a kind gift from Prof. Karl-Josef Dietz, Lehrstuhl für Biochemie und Physiologie der Pflanzen, Universität Bielefeld, Bielefeld, Germany) overnight at 4°C. The antibody was used at a 1∶1000 dilution. After washing with TBS (containing 0.1% Tween-20), membranes were incubated with horseradish peroxidase-conjugated goat anti-rabbit secondary antibody (Jackson ImmunoResearch Laboratories, Inc., West Grove, PA, USA) for 1 h at room temperature. Membrane-bound V-H^+^-ATPase was detected with SuperSignal West Pico Chemiluminescent Substrate (Thermo Fisher Scientific Inc., Rockford, IL, USA). The images were acquired with a ChemiDOC XRS instrument (Bio-Rad), and band intensity was analyzed with Quantity One-1D analysis software.

### Immunofluorescency

Immunolocalization of subunit E of V-H^+^-ATPase (VHA-E) was performed according to Golldack and Dietz with minor modifications [17]. In brief, leaf and root (within the root hair zone) tissues were fixed in 4% paraformaldehyde and embedded in OCT compound (Sakura Finetek, CA, USA). Then, 7 μm sections were cut using a Leica CM1950 cryostat (Leica Biosystems Nussloch GmbH, Heidelberger, Germany) and mounted on poly-L-Lys-coated microscopic slides. The slides were blocked with 1% BSA in phosphate-buffered saline (PBS; 150 mM NaCl, 5 mM KCl, 0.8 mM KH_2_PO_4_, 3.2 mM Na_2_HPO_4_, pH 7.2) for 15 min. The sections were incubated with rabbit anti-VHA-E antibody (1∶500 dilution) overnight at 4°C. After being washed twice with PBS, the sections were incubated with Alexa fluo-635 conjugated anti-rabbit secondary antibody (Molecular Probes, Eugene, OR) at 1∶500 dilution for 30 min. Parallel sets processed without primary antibody were used as the negative controls. Nuclei were stained with 4′,6′-diamidino-2-phenylindole (DAPI) (Molecular Probes, Eugene, OR). Microscopic images were obtained with a Leica TCS SP5 confocal scanning microscope (Leica Microsystems, Heidelberg GmbH, Mannheim, Germany).

### pH measurements

Vacuolar pH was determined using the pH-sensitive fluorescent dye BCECF AM (Molecular Probes, Eugene, OR) in accordance with Krebs et al [26]. Loading of the dye was performed by incubating the plantlets in 1/10 MS medium containing 0.5% sucrose, 10 mM Mes-KOH (pH 5.8) and 0.02% Pluronic F-127 (Molecular Probes, Eugene, OR), for 1 h at 22°C in the dark. The final concentration of BCECF AM was 10 μM. Dye loading was terminated by washing the plant material in 1/10 MS medium. Subsequently, root segments (within the root hair zone) were examined longitudinally with a Leica TCS SP5 confocal scanning microscope. The specimens were excited at 488 and 458 nm, and the emission was detected between 530 and 550 nm. Ratio images (488/458 nm) were generated using the Leica LAS AF Lite software. An average ratio was calculated and used to determine the pH value using a pH-ratio calibration.

For the pH-ratio calibration in situ, the plantlets were incubated in pH equilibration buffers to equilibrate the vacuolar pH to that of the externa1 solution [30]. The equilibration solution contained 50 mM Mes-BTP (pH 5.2–6.4) or 50 mM Hepes-BTP (pH 6.8–7.6), 50 mM ammonium acetate and 450 mM sorbitol. The mean ratio values were obtained by measuring three plantlets at each pH and were used to generate the calibration curve.

### Statistical analysis

Data are presented as the means ± SEM of three replicates. All data were subjected to one-way ANOVA analysis using SigmaStat3.5 software, and significant differences among treatments were calculated with Duncan's multiple range tests (*P* = 0.05).

## Results

### Phenotypes of salt tolerance

After 5 days of exposure to NaCl, the phenotypes of *B. papyrifera* plantlets were recorded (Fig. S1). Plants treated with 50 mM and 100 mM NaCl grew well without obvious symptoms of salt injury. However, plants treated with 150 mM NaCl exhibited symptoms of salt injury, including chlorosis of leaves and leaf tip necrosis. These findings indicated that *B. papyrifera* could tolerate up to 100 mM NaCl.

### Effects of NaCl stress on V-H^+^-ATPase and V-H^+^-PPase hydrolytic activity in the leaves and roots of *B. papyrifera*

**Figure 1:**
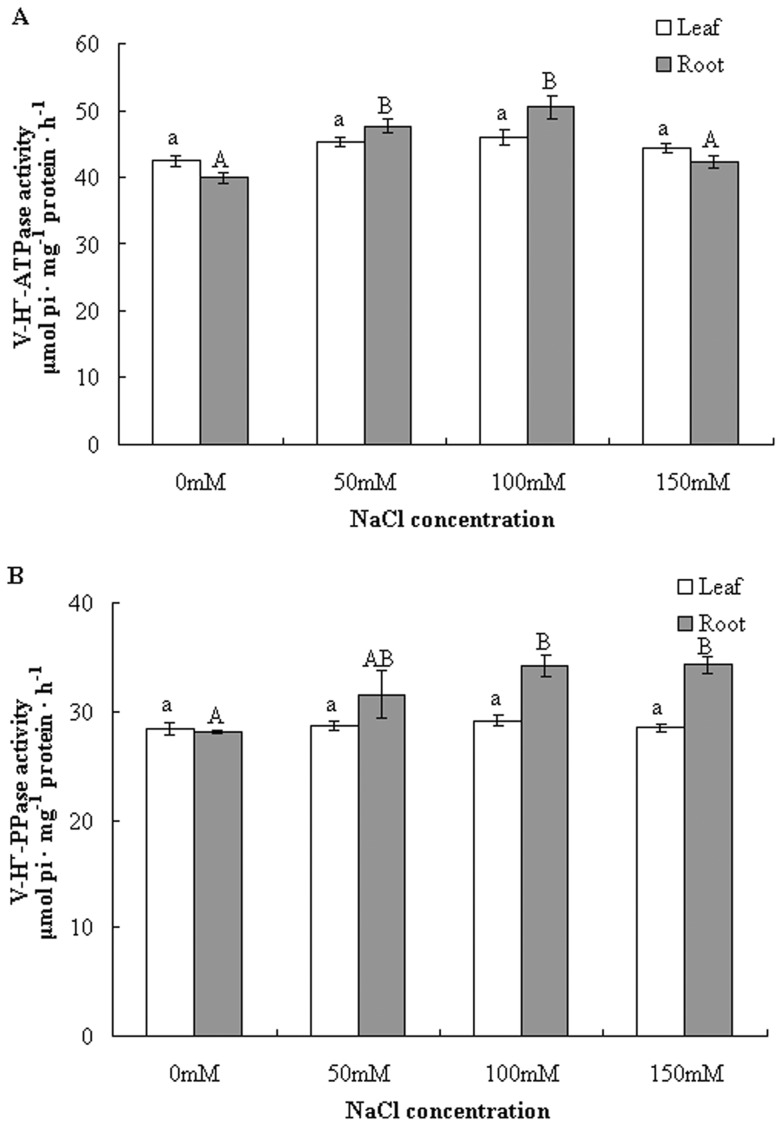
Effect of NaCl stress on the hydrolytic activity of V-H^+^-ATPase (A) and V-H^+^-PPase (B). Tonoplasts isolated from the leaves and roots of control and salt stressed plants were used. V-H^+^-ATPase activity was determined by measuring the amount of inorganic phosphate released in the presence and absence of concanamycin A, and V-H^+^-PPase activity was measured in the presence and absence of KCl. The results are presented as the means ± SEM of three replicates, and different letters indicate significant differences among treatments (*P*<0.05).

Because previous studies have shown that salt stress induced enhancement of V-H^+^-ATPase and H^+^-PPase activities in some plant species [4], [11], [12], [14], we first determined changes in tonoplast H^+^-ATPase and H^+^-PPase activity in the woody plant *B. papyrifera* under NaCl stress. Our preliminary experiment showed that 100 nM concanamycin A resulted in 83% inhibition of V-H^+^-ATPase hydrolytic activity, indicating that the isolated membrane vesicles were enriched in tonoplasts without significant contamination by other cellular membranes. A distinct activity profile for V-H^+^-ATPase was observed in the leaves and roots in response to NaCl. In the leaves, NaCl only induced a slight increase in V-H^+^-ATPase activity, whereas it markedly stimulated V-H^+^-ATPase activity in the roots. ATP hydrolysis activity was increased by 6.8%, 8.2% and 4.5% at the 50 mM, 100 mM and 150 mM NaCl treatments, respectively, in the leaves, relative to those of untreated plants (Fig. 1A). In contrast, in the roots, salt-induced stimulation of hydrolytic activity reached 19.1% and 26.1% at 50 mM and 100 mM NaCl, respectively, while 150 mM NaCl treatment did not induce a significant increase in V-H^+^-ATPase activity (5.8%) (Fig. 1A).

The alterations in H^+^-PPase activity induced by salt stress were very similar to that of H^+^-ATPase. Tonoplast H^+^-PPase activity remained relatively constant under different concentrations of NaCl stress in the leaves (Fig. 1B). In comparison to the leaves, salt stress led to a sharp increase in H^+^-PPase activity in the roots. The increase in H^+^-PPase activity was more evident at 100 and 150 mM NaCl (21.6% and 22.1%, respectively) (Fig. 1B).

### NaCl treatment affected proton transport activity of tonoplast H^+^-ATPase and vacuolar pH

**Figure 2:**
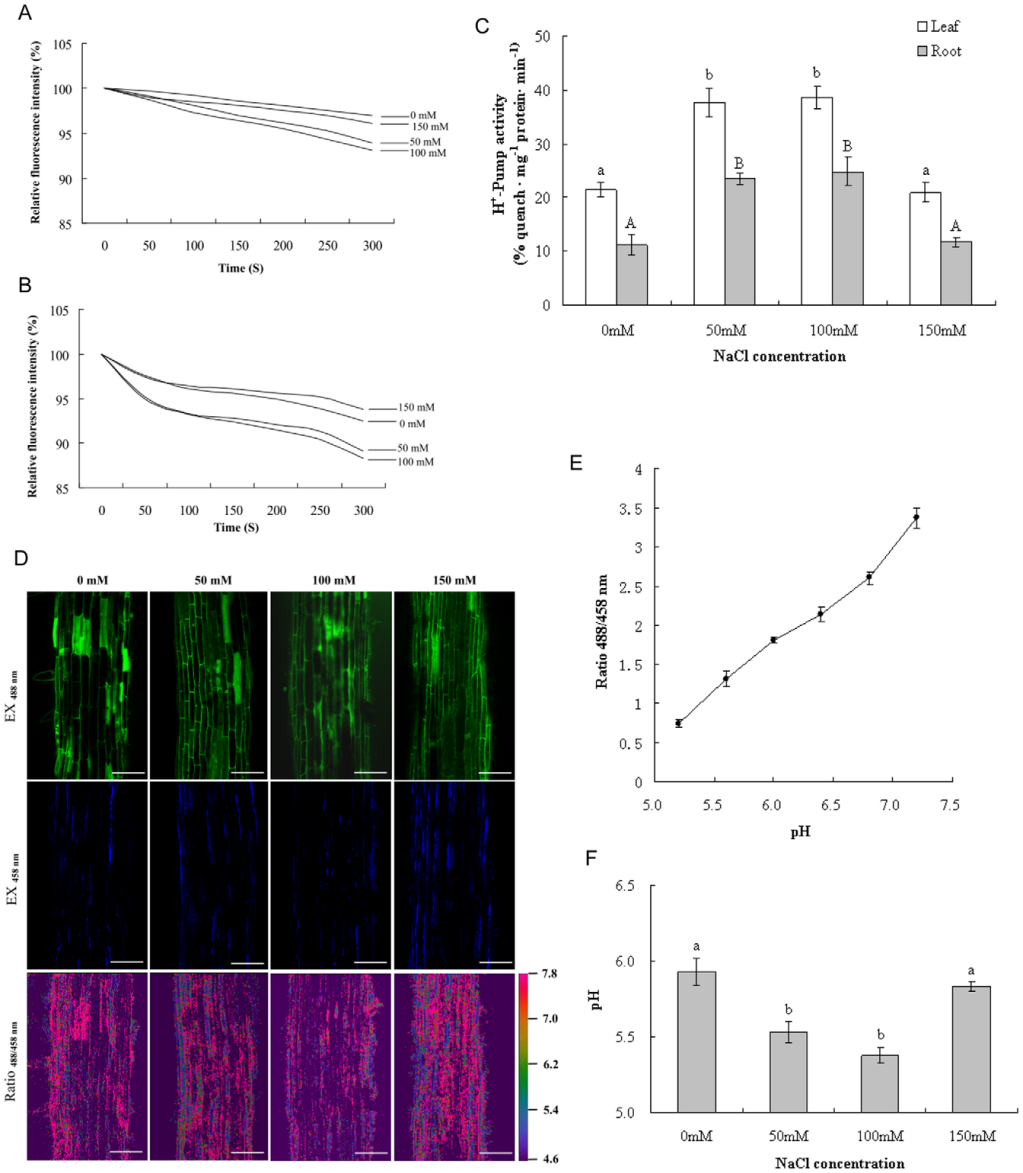
NaCl stress-induced changes in H^+^-Pump activity of V-H^+^-ATPase (A–C) and vacuolar pH (D–F). Fluorescence quenching of ACMA in the root (A) and leaf (B) and changes in H^+^-Pump activity of V-H^+^-ATPase (C) were presented. (D) Confocal images of root cell vacuoles loaded with BCECF AM. Pseudocolor in the ratio image enhances visualization of dye distribution and fluorescence intensity of the dye. (Scale bar  = 100 μm) (E) In situ pH-ratio calibration curve. The calibration curve was obtained by plotting the fluorescence ratios (488/458 nm) against the pH of the equilibration buffers. (F) Changes in vacuolar pH with different concentrations of NaCl treatment.

The proton transport activity of V-H^+^-ATPase was measured as the fluorescence quenching of ACMA. As shown in Fig. 2A and C, fluorescence quenching in the roots of control plantlets was 11.2%, while it reached 23.4% at 50 mM NaCl, an increase of 108.9%. Proton transport activity exhibited 122.3% stimulation when the NaCl concentration rose to 100 mM. However, proton transport activity showed only a slight change in comparison to the control when NaCl reached 150 mM. The results also showed that proton transport activity in the leaves was activated by NaCl treatment in a similar manner to that in the roots (Fig. 2 B and C). Proton transport activity was increased by 76.2% and 80.4% at 50 mM and 100 mM NaCl, respectively. In contrast, 150 mM NaCl only induced a minimal change in proton transport activity.

To determine the influence of NaCl on vacuolar pH, the pH-sensitive fluorescent dye BCECF was loaded in the plant roots for in situ pH measurements. As indicated in Fig. 2D–F, significant pH changes were observed after exposure to NaCl. In control roots the vacuolar pH was 5.9; in contrast, 50 mM and 100 mM NaCl treatment resulted in vacuolar acidification by 0.4 and 0.5 pH units, respectively. In addition, 150 mM NaCl caused only a slight decrease in vacuolar pH, from 5.9 to 5.8, compared with the control.

### Expression of V-H^+^-ATPase subunit genes in the leaves and roots is differentially regulated under NaCl stress

**Figure 3:**
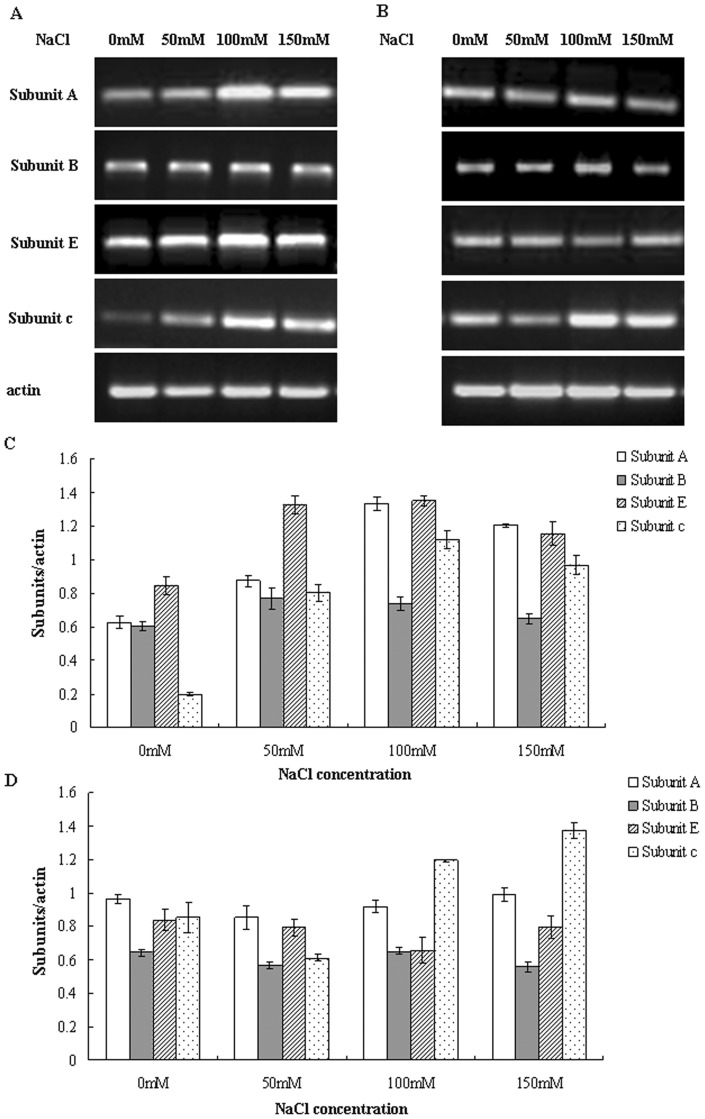
RT-PCR analysis of the expression of genes for V-H^+^-ATPase subunits A, B, E and c. RNA was isolated from roots (A) and leaves (B), and the transcript levels of V-H^+^-ATPase subunits were analyzed by RT-PCR. PCR products of the roots (C) and leaves (D) were quantified using Quantity One-1D software. RT-PCR results were normalized to their respective actin bands and expressed as fold changes.

To examine whether the expression of the genes encoding V-H^+^-ATPase could be modulated by salinity, the transcript levels of subunits A, B, E and c were determined using RT-PCR. Our results indicated that the relative mRNA levels of subunits A, B, E and c were differentially regulated by salinity in the leaves and roots (Fig. 3). As shown in Fig. 3A and C, transcript levels of subunits A and E in the roots were significantly enhanced by salt stress. There was 1.4-, 2.1- and 1.9-fold more mRNA for subunit A under the conditions of 50, 100 and 150 mM NaCl, respectively, in comparison to control roots. Transcript levels of subunit E were increased by 1.5-, 1.6- and 1.4-fold, respectively, when exposed to the same concentrations of NaCl. However, the transcript levels of subunits A and E did not change significantly in salt-stressed leaves (Fig. 3B and D). Moreover, the mRNA levels for subunit B showed only slight changes in both leaves and roots during salt treatment. Consistent with previous studies [31], [32], we observed an enhancement in transcript levels of subunit c with NaCl treatment, especially at 100 and 150 mM NaCl, which induced 5.6- and 4.9-fold increases in the mRNA level, respectively, in the roots, while mRNA expression was elevated by 1.4- and 1.6-fold, respectively, in the leaves.

### Expression of V-H^+^-ATPase subunit E protein in the roots is enhanced by NaCl stress

**Figure 4:**
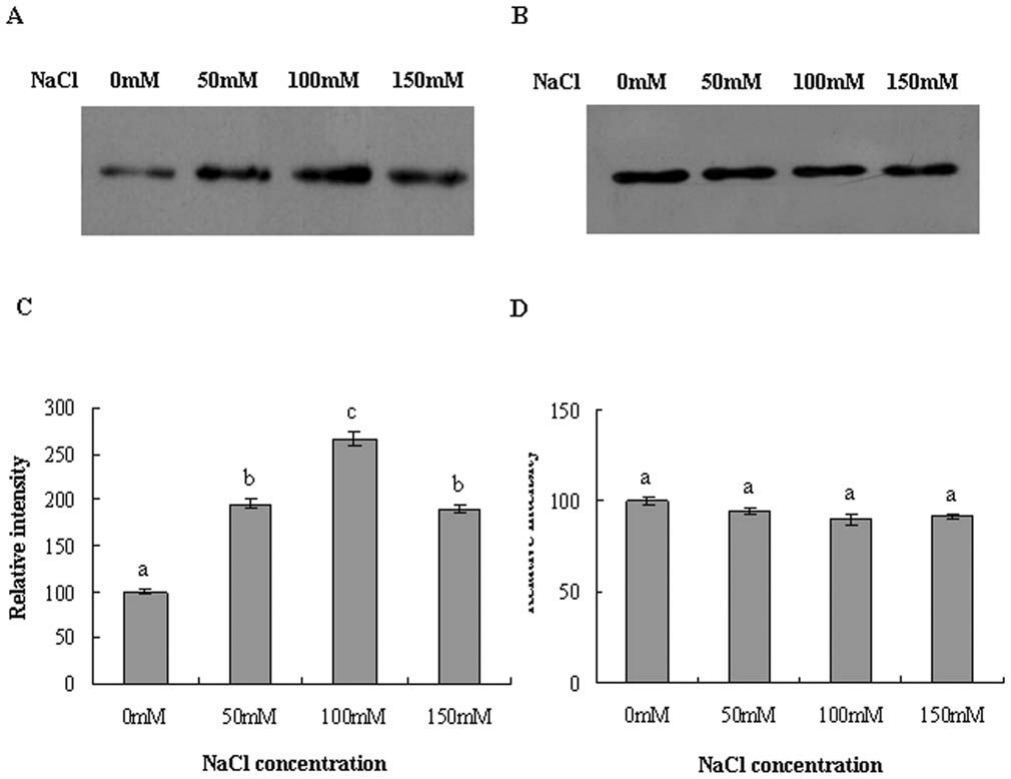
Western blot analysis of V-H^+^-ATPase subunit E protein expression in response to NaCl stress. Immunostaining was performed using rabbit anti-VHA-E antibody. Western blots of tonoplast vesicles from roots (A) and leaves (B) are presented. Subunit E protein levels in the roots (C) and leaves (D) were quantified. The protein level of subunit E in the control was set to 100, and the protein levels of NaCl stressed plants were compared with control.

We next performed immunodetection of V-H^+^-ATPase subunit E by Western blot analysis. As shown in Fig. 4A, in parallel with the increase in the level of subunit E transcripts, a dramatic elevation in the protein level of subunit E in the roots was observed following NaCl treatments. Densitometric analysis revealed 95.9%, 166.8% and 89.9% induction in the protein level with 50, 100 and 150 mM NaCl, respectively, in the roots (Fig. 4C). In contrast, NaCl treatments failed to stimulate the expression of V-H^+^-ATPase protein in the leaves, consistent with the RT-PCR findings (Fig. 4B, D).

### Immunolocalization of V-H^+^-ATPase subunit E in leaves and roots

**Figure 5:**
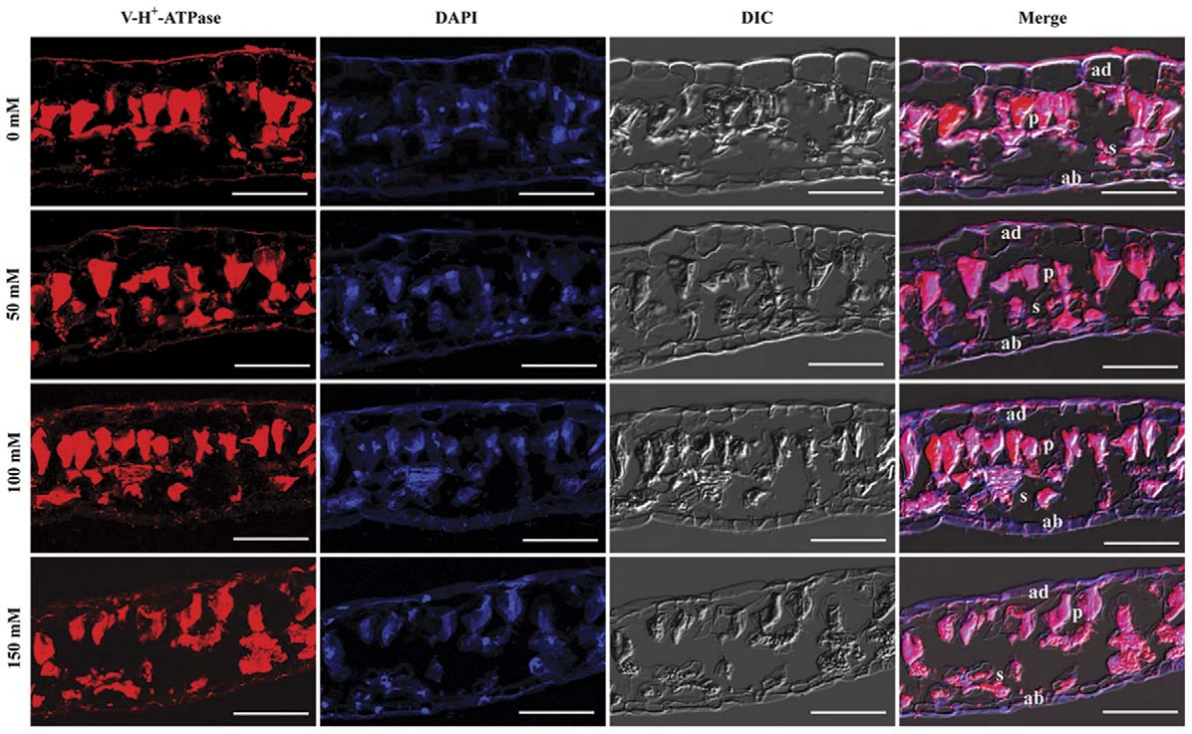
Immunolocalization of V-H^+^-ATPase subunit E in the leaves of *B. papyrifera*. V-H^+^-ATPase subunit E was stained red with rabbit anti-VHA-E antibody and the nuclei were stained blue with DAPI. The merged images of VHA-E, nuclei and the DIC image are also presented. DIC, differential interference contrast; ad, adaxial epidermis; ab, abaxial epidermis; p, palisade tissue; s, spongy parenchyma. Scale bar  = 50 μm.

**Figure 6:**
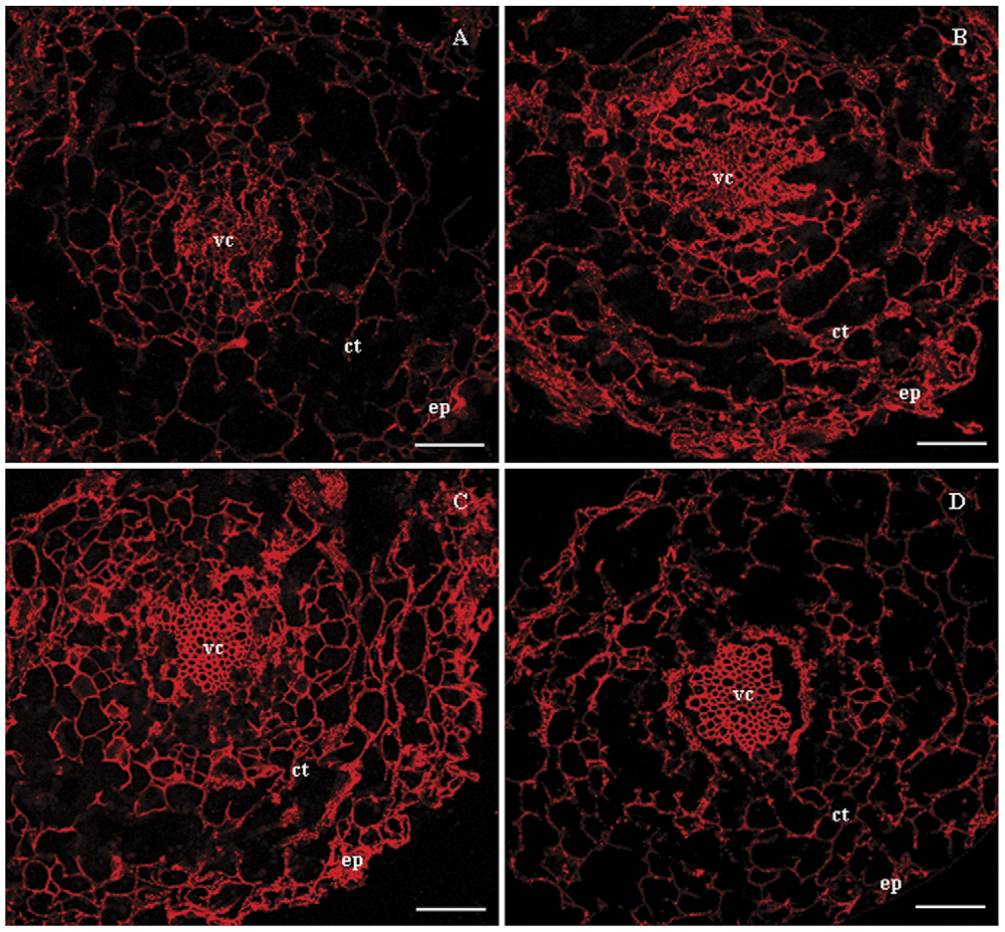
Distribution of V-H^+^-ATPase subunit E protein in root tissues of *B. papyrifera* grown under NaCl stress. (A) Control, (B) 50 mM NaCl treated plants, (C) 100 mM NaCl treated plants and (D) 150 mM NaCl treated plants. Localization of VHA-E was examined by immunofluorescency using rabbit anti-VHA-E antibody. ep, epidermis; ct, cortex; vc, vascular cylinder. Scale bar  = 50 μm.

Cytolocalization of subunit E in leaf and root cross-sections was conducted using immunofluorescency. In control and NaCl-stressed leaves, subunit E was found in leaf mesophyll cells of both palisade tissue and spongy parenchyma (Fig. 5). The signal intensity of subunit E after each treatment was similar, indicating that NaCl had little effect on the distribution of subunit E in leaf tissues. Signals for subunit E were detected in all cell types of the epidermis, cortex and vascular cylinder in control roots, with the strongest expression present in the vascular cylinder (Fig. 6A). When exposed to 50 mM NaCl, the signal intensity of subunit E was clearly enhanced in all tissues (Fig. 6B). The expression of subunit E was further enhanced with elevated NaCl concentration, especially in the vascular cylinder, where a large amount of subunit E protein accumulated (Fig. 6C). Moreover, at 150 mM NaCl the signal intensity of subunit E was lower in the epidermis and cortex compared with the 100 mM NaCl treatment; however, it was still stronger than that in the control roots (Fig. 6D), and the accumulation of subunit E protein in the vascular cylinder was not decreased.

## Discussion

Sequestration of Na^+^ into the vacuole has been considered one of the most effective ways to maintain intracellular ion homeostasis [4]. The exclusion of Na^+^ from the cytosol by the vacuole is driven by an electrochemical gradient in the membranes generated by V-H^+^-ATPase and V-H^+^-PPase. Thus, regulation of V-H^+^-ATPase might play an essential role in plant salt tolerance. In the present study, we observed enhancements in V-H^+^-ATPase hydrolytic and H^+^ pumping activities in the roots of *B. papyrifera* in response to NaCl stress. Moreover, transcript analysis of subunits A, B, E and c of V-H^+^-ATPase showed an increase in the expression of subunits A, E and c gene. And Western blot analysis using the antibody to V-H^+^-ATPase subunit E revealed an elevation in the protein level of subunit E. This NaCl-induced stimulation of V-H^+^-ATPase may be associated with transcriptional activation of subunits A, E and c and up-regulation of the protein level of subunit E.

An array of evidence has demonstrated that the activity of V-H^+^-ATPase is increased in most plants in response to salt stress [12], [13], [15]. However, contrary findings have also been documented, such as data from wheat roots under severe NaCl stress, where V-H^+^-ATPase activity was significantly depressed [33]. Moreover, a recent study showed that V-H^+^-ATPase activity increased a short time (24 h) after treatment with NaCl, whereas the activity decreased with prolonged exposure time (4 d and 8 d) [14]. In our study, V-H^+^-ATPase activity was stimulated in the roots, but only slight changes were observed in the leaves of *B. papyrifera*. Together this suggests that the activity of V-H^+^-ATPase responds differently to salinity in different plant species, and changes in V-H^+^-ATPase activity are concentration and time dependent.

*B. papyrifera* grew well in 50 mM and 100 mM NaCl, whereas when NaCl reached 150 mM, chlorosis of the leaves and leaf tip necrosis were observed. Consistent with these phenotypes, increases in the H^+^ pumping activity of V-H^+^-ATPase in leaves and roots from 50 mM and 100 mM NaCl treated plants were observed. In contrast, no obvious changes in the H^+^ pumping activity of V-H^+^-ATPase were detected at 150 mM NaCl. Meanwhile, acidification of vacuoles occurred, paralleling the increase in H^+^ pumping activity. Vacuolar pH was decreased by 0.4–0.5 pH units compared to control plants.

It has been suggested that changes in plant V-H^+^-ATPase activity occur in parallel to alterations in transcript levels and/or the amounts of different protein subunits of V-H^+^-ATPase after exposure to salinity stress. In this report, we analyzed the effects of NaCl exposure on the gene expression of subunits A, B, E and c and the protein levels of subunit E by RT-PCR and Western blot analysis. These revealed that salinity triggered a tissue-specific expressional response in *B. papyrifera* plantlets. A coordinated up-regulation of the mRNA levels for subunits A, E and c was noticed in the roots but not in the leaves of plants exposed to NaCl stress. This increase in mRNA levels was in parallel with the augmented V-H^+^-ATPase activity, suggesting the increased transcript levels may be partially responsible for the stimulation of V-H^+^-ATPase activity. Coordinated up-regulation of V-H^+^-ATPase subunits has also been shown in other plant species, including halotolerant sugar beet [19] and the common ice plant [34], [35]. Consistent with the enhancement of subunit E mRNA expression, an increase in its protein level occurred in the roots of salt-exposed *B. papyrifera*, indicating that the increase in protein expression may also be involved in the regulation of V-H^+^-ATPase activity.

In addition to translational regulation of V-H^+^-ATPase activity, some other mechanisms by which V-H^+^-ATPase activity may be regulated have been proposed. A recent study provided evidence that a WNK kinase, AtWNK8, could phosphorylate subunit C of V-H^+^-ATPase, indicating post-translational modifications were also involved in the regulation of V-H^+^-ATPase activity [36]. Moreover, the Ser/Thr kinase SOS2 was reported to promote salt tolerance by interacting with V-H^+^-ATPase and up-regulating its transport activity [9]. More recent research has found that the Cdc42 effector Ste20 stimulates V-H^+^-ATPase activity by forming a complex with Vma13, a regulatory subunit of V-H^+^-ATPase [37]. Several reports have suggested that V-H^+^-ATPase activity may also be modulated by assembly-disassembly of the V_1_ and V_0_ sectors [38], [39]. Additionally, changes in the lipid microenvironment of the vacuolar membrane may account for the regulation of V-H^+^-ATPase activity because it was reported that alterations in the membrane lipid composition and structure were associated with modulation of tonoplast transport proteins [40], [41]. Whether these mechanisms are involved in the regulation of V-H^+^-ATPase activity in *B. papyrifera* needs further investigation.

Altogether, we have shown the differential and tissue-specific expression of V-H^+^-ATPase subunits in response to salt stress. This indicates that the enhanced expression of V-H^+^-ATPase subunit E in the roots may confer salt tolerance to the woody plant *B. papyrifera*. These findings may provide insights into understanding the salt resistance of plants.

## Supporting Information

Figure S1 **Phenotype of** ***Broussonetia papyrifera*** **grown under different concentrations of NaCl.** (A) Whole plants of control and NaCl treated *B. papyrifera*. (B) Leaves from the corresponding plants. Note the leaf tip chlorosis and necrosis in 150 mM NaCl treated plants.
(TIF)
