# Supplementary figures and images for: Enhanced Expression of Vacuolar H+-ATPase Subunit E in the Roots Is Associated with the Adaptation of Broussonetia papyrifera to Salt Stress
Source: PLoS One. 2012 Oct 25;7(10):e48183. doi: 10.1371/journal.pone.0048183 (PMC3485061; doi:10.1371/journal.pone.0048183)

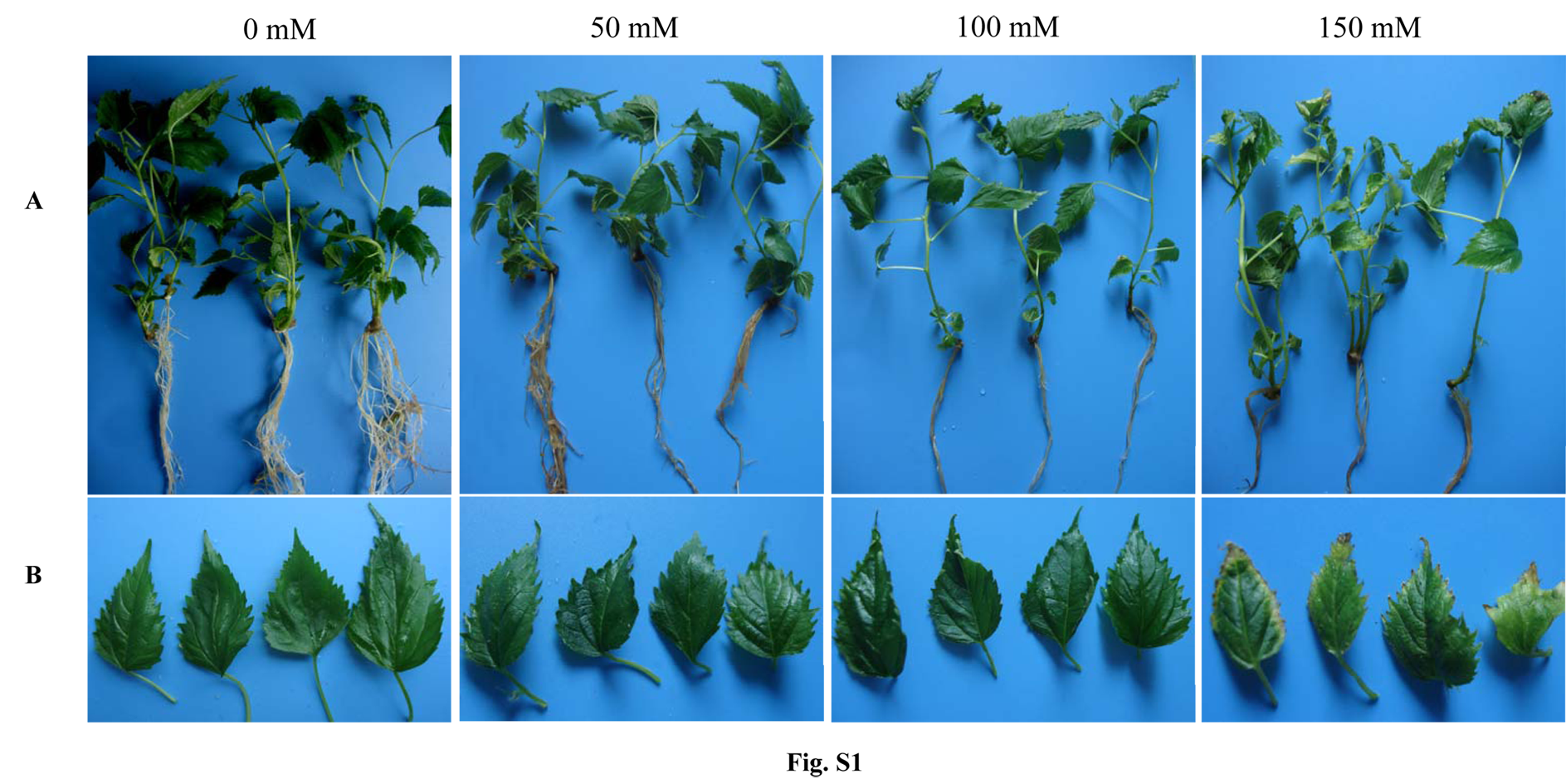

Supplement: Figure S1 — Phenotype of Broussonetia papyrifera grown under different concentrations of NaCl. (A) Whole plants of control and NaCl treated B. papyrifera. (B) Leaves from the corresponding plants. Note the leaf tip chlorosis and necrosis in 150 mM NaCl treated plants. (TIF) [file pone.0048183.s001.tif]
